# Polymorphisms and dental age in non-syndromic cleft lip and palate: a cross-sectional study

**DOI:** 10.1186/s12887-025-05444-8

**Published:** 2025-01-30

**Authors:** Gabriela Fonseca-Souza, Lhorrany Alves-Souza, Maria Angélica Hueb de Menezes-Oliveira, Nikolaos Daratsianos, Svenja Beisel-Memmert, Christian Kirschneck, Rafaela Scariot, Juliana Feltrin-Souza, Erika Calvano Küchler

**Affiliations:** 1 Department of Stomatology, Federal University of Paraná, Av. Prefeito Lothário Meissner 632, Jardim Botânico, Curitiba, 80210-170 Paraná Brazil; 2 Department of Biomaterials, University of Uberaba, Av. Nenê Sabino 1801, Bairro Universitário, Uberaba, 38055-500 Minas Gerais Brazil; 3 Department of Orthodontics, University Hospital Bonn, Medical Faculty, Welschnonnenstr. 17, 53111 Bonn, Germany

**Keywords:** Cleft lip, Cleft palate, Tooth abnormalities, Anodontia, Age determination by teeth, Polymorphism, genetic

## Abstract

**Background:**

Children with non-syndromic cleft lip with or without palate (CL ± P) may present alterations in dental development. The purpose of this cross-sectional study was to compare the dental age (DA) between children with and without CL ± P, and whether single nucleotide polymorphisms (SNPs) in genes encoding growth factors are associated with DA variations.

**Methods:**

Children aged between 5 and 14 years with and without CL ± P were recruited to participate in this study. DA was evaluated by calibrated examiners (kappa > 0.80) using the method proposed by Demirjian et al. (1973). Genomic DNA was extracted from buccal cells, and SNPs in Epidermal Growth Factor (*EGF*) – rs4444903 and rs2237051, Epidermal Growth Factor Receptor (*EGFR*) – rs2227983 –, Transforming Growth Factor Beta 1 (*TGFB1*) – rs1800470 and rs4803455 –, and Transforming Growth Factor Beta Receptor 2 (*TGFBR2*) – rs3087465 – were genotyped by real-time polymerase chain reactions using the TaqMan assay. The Student T-test was used to compare the variations in DA between the phenotypes “with CL ± P” and “without CL ± P”, and the ANOVA two-way test was performed to compare the variations in DA among the genotypes (α = 0.05). A *post-hoc* analysis was performed using Bonferroni correction.

**Results:**

Two hundred and nine (*n* = 209) children (100 with CL ± P and 109 without CL ± P) with a mean chronological age of 8.66 years – standard deviation (SD) = 1.92 – were included. The group with CL ± P demonstrated a significantly delayed DA (mean=-0.23; SD = 0.71) compared to the group without CL ± P (mean=-0.01; SD = 0.88) (*p* = 0.049). Genotype distributions were in Hardy-Weinberg equilibrium. The SNP rs4803455 in *TGFB1* was significantly associated with DA variations in children without CL ± P (*p* < 0.01). In the group with CL ± P, no significant differences in DA were observed among the genotypes.

**Conclusion:**

Children with CL ± P presented delayed DA compared with children without CL ± P. The SNP rs4803455 in *TGFB1* is associated with variations in DA in children without CL ± P.

**Supplementary Information:**

The online version contains supplementary material available at 10.1186/s12887-025-05444-8.

## Background

Non-syndromic oral clefts are among the most common congenital anomalies globally, arising from developmental disruptions in the development of the primary and secondary palate [1], being classified into cleft lip (CL), cleft lip and palate (CLP), and cleft palate (CP). Since the lip and primary palate present different embryological origins from the secondary palate, CL and CLP are usually grouped as cleft lip with or without palate (CL ± P) [2].

Both genetic and environmental factors influence the formation of the primary and secondary palate and the teeth. Any disturbances in this process may result in craniofacial and/or dental abnormalities [3]. Several dental alterations are associated with oral clefts, especially with CL ± P [4]. A previous systematic review reported that patients affected by these craniofacial defects tend to present delayed dental age (DA) compared to not affected patients [5]. DA is a biological marker used in the evaluation of the growth status of a child, presenting applicability in the forensic field as well as in dental clinical practice [6]. During childhood, patients with CL ± P need numerous interventions in the craniofacial region, including orthodontic and surgical procedures [7], and the maturation status is an important indicator for choosing the best time to perform these treatments and achieve good outcomes [5]. Thus, DA may be used as an initial tool for the estimation of a child’s growth status [8].

Genes encoding growth factors present pleiotropic activity, playing a role in both dental and craniofacial formation. The Epidermal Growth Factor (EGF) and its receptor (EGFR) are required for adequate palatal fusion [9], dental development and eruption [10–12]. Transforming Growth Factor 1 (TGFB1) has a function during cell proliferation and growth of the palatal shelves [13], as well as during odontoblast and ameloblast differentiation [14, 15]. TGFB1 acts by binding to the Transforming Growth Factor Receptor 2 (TGFBR2), which is expressed in the palate during its fusion [16], in the embryonic dental pulp, and in the root pre-odontoblasts [17, 18]. Single nucleotide polymorphisms (SNPs) in genes encoding growth factors have been reported to be associated with alterations in the craniofacial region, including CL ± P (rs4444903 in *EGF*) [19], mandibular retrognathia (rs3087465 in *TGFBR2*), and variations in tooth size (rs4444903 in *EGF* and rs1800470 in *TGFΒ1*) [20].

Despite some studies investigated the association between DA and SNPs in the general population [6, 21, 22], there is a gap in understanding how these genetic variations influence DA in children with CL ± P. This knowledge gap limits the understanding of how these genetic factors contribute to the dental development of these children, who often face unique developmental challenges. Thus, this study aims to compare DA between children with and without CL ± P, and to evaluate the association between SNPs in genes encoding growth factors and DA variations.

## Materials and methods

### Ethical approval

This comparative cross-sectional study was approved by the Committee for Ethics in Research in Human Beings of the Health Sciences of the Federal University of Paraná (protocol number 3.752.172) and by the Committee for Ethics in Research of the State Health Department of Paraná (protocol number 5.100.185), both located in Curitiba, Paraná, Brazil. The assent and consent were obtained from the children and their legal guardians, respectively. This study is being reported according to the Strengthening the Reporting of Genetic Association Study Statement Checklist (STREGA) [23]. Data collection was performed from January 2022 to August 2023.

### Eligibility criteria and sample size calculation

Children with CL ± P aged between 5 and 14 years old were recruited from a reference center for treatment of craniofacial deformities located in Brazil (Centro de Atendimento Integral ao Fissurado Labiopalatal – CAIF –, Curitiba, Paraná, Brazil). This center was the first one located in southern Brazil destined for the treatment of craniofacial anomalies, providing medical and dental care, genetic consultation, as well all psychological and social support [24]. The cleft type was classified according to the clinical examination and confirmed by medical records. Cases of CP were not included due to the distinct embryologic origin of this cleft type [2]. A comparison group of children aged between 5 and 14 years without CL ± P, confirmed by clinical examination, was selected from the dental clinics at the Federal University of Paraná (UFPR, Curitiba, Paraná, Brazil). Children with syndromes, craniofacial anomalies other than oral clefts, or without panoramic radiography were excluded.

The sample size was determined according to the association data between orofacial clefts and tooth abnormalities [4]. The calculation was based on a power of 80%, a confidence interval of 95% (95%CI), an odds ratio (OR) of 3.14, and a rate of tooth abnormalities between individuals without oral clefts of 38.8%. Considering a loss of 20%, the final estimative of sample size should be a minimum of 100 and a maximum of 120 children per group. The participants were selected for convenience, with patients present at the recruitment site being invited to participate.

### DA assessment

Panoramic radiographs obtained from children’s dental records were evaluated by two examiners to assess DA [25]. The examiners rated the permanent left mandibular teeth (excluding the third molar) on a scale of A to H according to the dental mineralization stage. When a permanent tooth on the left side of the mandible was missing, the contralateral permanent tooth on the right side was evaluated. Children with one or more teeth missing bilaterally were excluded. Previously to the panoramic radiograph evaluation, the examiners, two pediatric dentists (G.F.S and L.A.S), went through a training and calibration process. This process started with a theoretical discussion of Demirjian’s method [25] with a reference examiner with experience in this methodology. The calibration included assessment of 20 panoramic radiographs presenting various stages of teeth mineralization. The examiners evaluated each radiograph independently and repeated the evaluations after a one-week interval to calculate intra-examiner reliability. To assess inter-examiner reliability, the scores were compared with those of the reference examiner. The intra and inter-rate kappa coefficients were 0.90 and 0.82, respectively.

DA was calculated using the Dental Age mobile app [26], which, according to the child’s sex, converts the rating given for the seven teeth evaluated into a DA value derived from tables provided by Demirjian et al. [25]. The variations in DA were determined by a delta calculated considering the difference between the DA and chronological age (CA) (DA-CA). Positive, negative, and null values mean that the child tends to have advanced, delayed, and normal dental age, respectively [22].

It is reported in the literature that Demirjian’s method overestimates DA in relation to CA [27]. Thus, we evaluated the correlation between DA and CA in our sample using the Spearman correlation coefficient test. A positive strong correlation was found between the CA and DA in both groups – with and without CL ± P (*p* < 0.01) (Table 1), confirming the applicability of Demirjian’s method in the present study population.

**Table 1:** Correlation between CA and DA according to the group (*n* = 209)

	DA	Groups	
		With CL ± *P*	Without CL ± *P*
CA	r*	0.886	0.905
	p value	**< 0.01**	**< 0.01**

*Spearman rank-order correlation coefficient. Bold indicates statistically significant results

Abbreviations: CL ± P – Cleft lip with or without palate; CA – Chronological age; DA – Dental age

### DNA extraction and genotyping analysis

DNA was extracted from buccal cells collected by a 5 ml mouthwash of saline solution [28]. DNA’s concentration and purity were determined by spectrophotometry (Nanodrop 2000, Thermo Scientific, Wilmington, DE, USA). Genotyping was performed by real-time polymerase chain reaction (PCR) (Thermo Fisher Scientific, Waltham, Mass., USA). All the laboratory analyses were blinded to the patient’s condition.

The SNPs in *EGF (*rs2237051 and rs4444903), *EGFR (*rs2227983), *TGFΒ-1 (*rs1800470 and rs4803455), and *TGFΒR-2 (*rs3087465 and rs764522) were selected based on their minor allele frequency (MAF) and their function. The characteristics of the genes and selected SNPs are presented in Table 2. The genotype success rate is presented in S1 Table.

**Table 2:** Characteristics of the selected genes and polymorphisms

Gene	Gene’s role		SNP’s characteristics			
	Palate development	Dental development	ID	Base change	Function	Global MAF
*EGF*	• Required for epithelial cell growth and differentiation [9, 14, 15]. • Participates in the degeneration of the medial edge epithelial cells during the secondary palate formation [9, 14, 15].	• Inhibits the morphogenesis and cellular differentiation of dental tissues during embryogenesis [10]. • Has a role during tooth eruption [11, 12].	rs4444903	A > G	Non-Coding Transcript	0.424
			rs2237051	A > G	Missense	0.412
*EGFR*	• Required, for epithelial cell growth and differentiation [9, 14, 15]. • Participates in the degeneration of the medial edge epithelial cells during the secondary palate formation [9, 14, 15].	• Influences cell proliferation rate during odontogenesis, impacting the duration of this process [33].	rs2227983	A > G	Missense	0.264
*TGFB1*	• Plays a role in cell proliferation and growth of the palatal shelves [13]. • Regulates mesenchymal cell proliferation and extracellular matrix synthesis in the palate [13].	• Participates in the regulation of odontoblast differentiation [14]. • Participates in the differentiation of the enamel organ and initiation of matrix secretion [15].	rs4803455	C > A	Intron	0.480
			rs1800470	A > G	Missense	0.495
*TGFBR2*	• Plays a role in normal palatal fusion [16].	• Presents diverse functions in tooth development and pulp tissue repair [17]. • Plays a role in osteodentin formation [18].	rs764522	C > G	Upstream	0.253
			rs3087465	A > G	Upstream	0.248

Obtained from databases: http://www.thermofisher.com and http://www.ncbi.nlm.nih.gov

SNP – Single nucleotide polymorphism; ID – Identification; MAF – Minor allele frequency

### Statistical analysis

The dependent variable “DA” was evaluated as a continuous variable. The independent variables were assessed as categorical variables: the phenotype was dichotomized according to the group into “with CL ± P” and “without CL ± P” and the genotype was categorized according to the pair of alleles of each SNP. The Student T-test was used to compare the variations in DA between the phenotypes “with CL ± P” and “without CL ± P”. P-values less than 0.05 were considered statistically significant.

Regarding the genotyping data, Hardy-Weinberg equilibrium was tested by chi-square test (https://wpcalc.com/en/equilibrium-hardy-weinberg/). A two-way ANOVA was performed to analyze the effect of the independent variables “phenotype” and “genotype”, as well as the interaction between those variables on DA variation (α = 5%). To reduce the occurrence of false positives, Bonferroni correction was adopted at a significance level of 0.016. All the statistical analysis was carried out in the Statistical Package for Social Sciences 20.0 software (SPSS, Chicago, IL, USA).

## Results

**Fig. 1:**
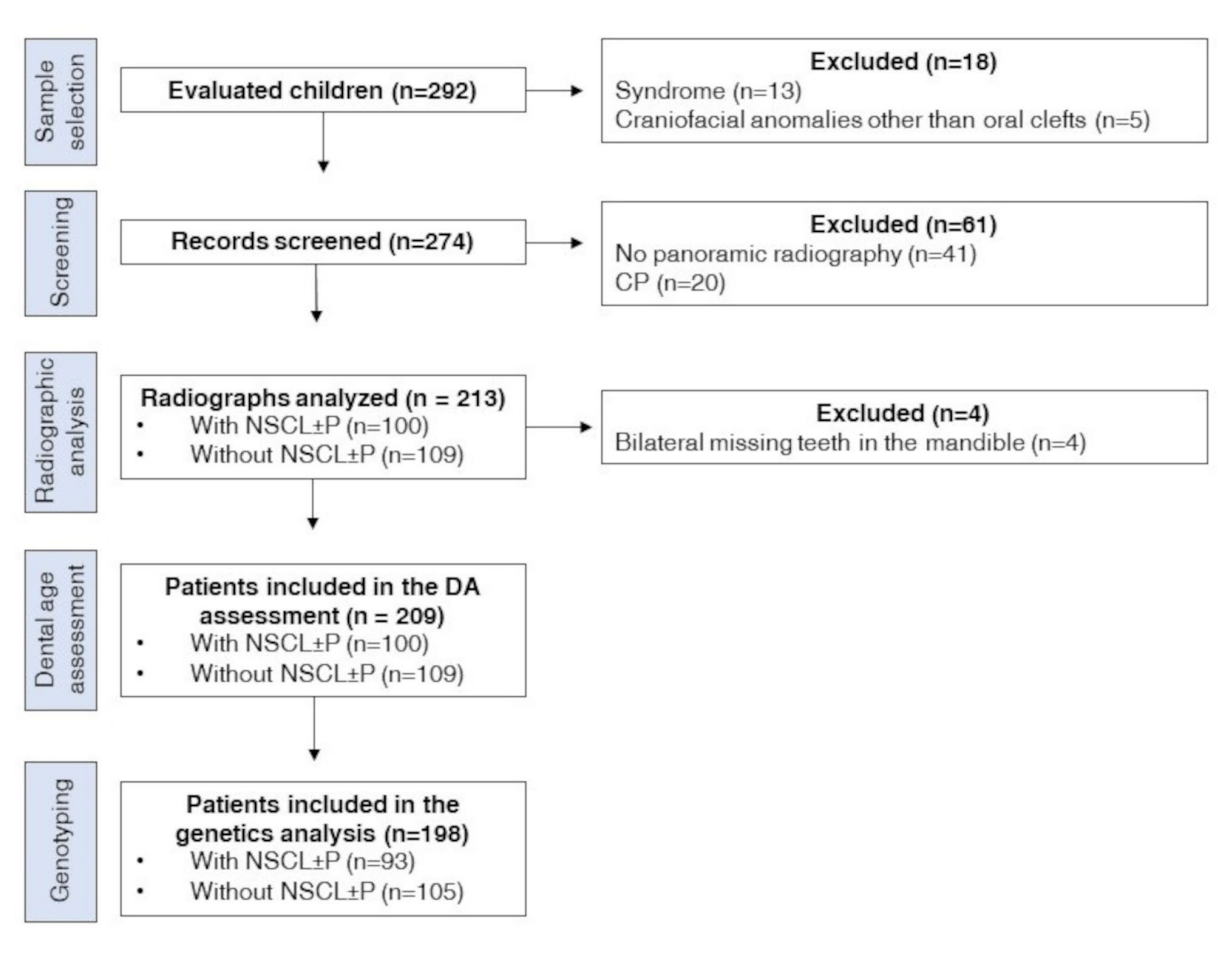
Flow diagram of the study

In total, 292 children were recruited and evaluated. Of those, 83 were excluded for the following reasons: the presence of syndrome (*n* = 13), the presence of other craniofacial anomalies than oral cleft (*n* = 5), the presence of CP (*n* = 20), no panoramic radiograph (*n* = 41), and bilateral missing teeth in the mandible (*n* = 4). The final sample comprised 100 children (61 boys and 39 girls) with CL ± P and 109 children (52 boys and 57 girls) without CL ± P. Figure 1 illustrates the flow diagram of the study.

In the group with CL ± P, 35 children (35%) presented CL and 65 (65%) had CLP. The mean chronological age of the total sample was 8.66 – standard deviation (SD) = 1.92. The group with CL ± P demonstrated a statistically significant delayed DA (mean = -0.23; SD = 0.71) compared to the group without CL ± P (mean = -0.01; SD = 0.88) (*p* = 0.049) (Table 3).

**Table 3:** Comparison of CA, DA, and delta according to the group and cleft type (*n* = 209)

Group	CA Mean (SD)	DA Mean (SD)	Delta (DA-CA) Mean (SD)
With CL ± P	8.52 (1.69)	8.29 (1.80)	-0.23 (0.71)
Without CL ± P	8.82 (2.12)	8.81 (1.96)	-0.01 (0.88)
p-value*	0.207	**0.036**	**0.049**

*Student t-test. Bold indicates statistically significant results

Abbreviations: CL ± P – Cleft lip with or without cleft palate; CA – Chronological age; DA – Dental age; SD – Standard deviation

DNA samples from 198 children (93 with CL ± P and 105 without CL ± P) were viable for genotyping analysis. The genotype distribution is presented in S1 Table. Table 4 shows the results obtained from two-way ANOVA, which was performed to analyze the effect of the independent variables “phenotype” and “genotype”, as well as the interaction between those variables on DA variation. The simple main effect test indicated that none of the SNPs evaluated presented a significant effect on DA variation (*p* > 0.05). However, when the interaction “phenotype-genotype” was considered, a statistically significant interaction between the effects of rs4803455 and the phenotype on DA variation was found (*p* < 0.01). *Post hoc* testing using Bonferroni correction showed that in the phenotype “without CL ± P”, DA was significantly advanced in children presenting the genotype AA in the rs4803455 (mean delta = 0.66; SD = 1.02) than those presenting CA (mean delta= -0.10; SD = 0.77) or CC (mean delta= -0.20; SD = 0.88) (*p* < 0.01). However, in the phenotype “with CL ± P”, no significant differences in DA were observed among the genotypes.

**Table 4:** Comparison of variations in DA according to the phenotype and genotype (ANOVA two-way; *n* = 198, 2024)

SNP (gene)	Genotype	DA mean (SD)		p-value*		
		With CL ± P	Without CL ± P	Phenotype effect	Genotype effect	Phenotype-genotype interaction
rs4444903 (*EGF*)	AA	-0.39 (SD = 0.16)	-0.13 (SD = 0.70)	**0.045**	0.521	0.723
	AG	-0.17 (SD = 0.17)	-0.03 (SD = 1.00)			
	GG	-0.32 (SD = 0.19)	-0.05 (SD = 0.75)			
rs2237051 (*EGF*)	AA	-0.28 (SD = 0.69)	-0.15 (SD = 0.80)	0.084	0.388	0.905
	AG	-0.16 (SD = 0.69)	-0.005 (SD = 0.96)			
	GG	-0.40 (SD = 0.70)	-0.13 (SD = 0.74)			
rs2227983 (*EGFR*)	AA	-0.85 (SD = 0.89)	-0.07 (SD = 1.05)	0.274	0.605	0.783
	AG	-0.23 (SD = 0.72)	0.08 (SD = 0.86)			
	GG	-0.30 (SD = 0.69)	-0.10 (SD = 0.88)			
rs4803455 (*TGFB1*)	CC	-0.05 (SD = 0.64)	-0.20 (SD = 0.88)	**0.002**	0.223	**0.002**
	CA	-0.35 (SD = 0.78)	-0.10 (SD = 0.77)			
	AA	-0.51 (SD = 0.44)	0.66 (SD = 1.02)			
rs1800470 (*TGFB1*)	AA	-0.45 (SD = 0.54)	0.19 (SD = 1.14)	0.058	0.646	0.068
	AG	-0.30 (SD = 0.82)	-0.08 (SD = 0.81)			
	GG	-0.007 (SD = 0.48)	-0.14 (SD = 0.81)			
rs764522 (*TGFBR2*)	CC	-0.18 (SD = 0.68)	0.007 (SD = 0.85)	0.111	0.324	0.774
	CG	-0.29 (SD = 0.76)	-0.14 (SD = 1.03)			
	GG	-0.67 (SD = 0.44)	-0.14 (SD = 0.53)			
rs3087465 (*TGFBR2*)	AA	-0.50 (SD = 0.83)	0.13 (SD = 0.91)	**0.029**	0.348	0.947
	AG	-0.32 (SD = 0.75)	-0.14 (SD = 0.85)			
	GG	-0.13 (SD = 0.63)	0.027 (SD = 0.93)			

* ANOVA two way is evaluating the effect of the independent variables “phenotype” and “genotype”, as well as the interaction between those variables on DA variation

Bold indicates statistically significant results

Abbreviations: CL ± P – Cleft lip with or without palate; SD – Standard deviation; *EGF* – Epidermal Growth Factor; *EGFR* – Epidermal Growth Factor Receptor; *TGFB1* – Transforming Growth Factor Beta 1; *TGFBR2* – Transforming Growth Factor Receptor 2

## Discussion

Individuals with oral clefts have a higher chance of presenting dental abnormalities than individuals without oral clefts [4]. In the present study, children with CL ± P presented delayed DA compared with children without CL ± P. A previous systematic review included 36 studies and reported that 32 of those found that patients with clefts tend to have a delay in dental development and eruption [5] compared to individuals without cleft. However, according to our knowledge, this is the first study investigating DA in CL ± P individuals and its genetic background.

The method proposed by Demirjian et al. (1973) is the most commonly used for DA assessment [29] and was adopted in the present study. In this method, the seven left mandibular teeth are evaluated [3]. The evaluation of mandibular teeth is preferred since, in the maxilla, the projection of bone structures over the teeth interferes with the assessment [5]. In cases of patients with CL ± P, the presence of the cleft defect and the frequent morphological dental abnormalities observed in the upper arch may also affect judgment [4]. The presence of dental abnormalities located outside the cleft area, including the ones in the mandible, suggests that similar genetic factors play a role in both dental and palatal development [30, 31]. Then, the variations in DA in patients with CL ± P observed in the present study, which were evaluated only considering lower teeth, may reinforce the shared genetic background between dental abnormalities and orofacial clefts.

Growth factors and their receptors, which are molecules that regulate several cellular processes, such as proliferation, differentiation, migration, or apoptosis [32] are reported to be involved in odontogenesis and palatogenesis [9–11, 13–17, 33]. The present study observed the effect of SNPs in growth factors encoding genes and the presence of CL ± P on DA variation. Among the SNPs in growth factors encoding genes evaluated here, only the rs4803455 in *TGFB1* was associated with variations in DA in children without CL ± P. In this group, children with the genotype AA in this SNP presented an advanced DA compared to those with the genotype CA or CC. This SNP is an intron variant, which does not have a coding function but possibly has a role in alternative splicing and gene expression regulation, messenger RNA transport, and chromatin assembly [34]. In the literature, this SNP was associated with different health conditions, including rheumatoid arthritis [35, 36], coronary heart disease [37], and lung cancer [38]. Regarding dental and craniofacial phenotypes, the evidence is scarce. Only one study investigated the role of rs1800470, finding an association between this SNP and variations in dental size [20]. Here is possible to suggest that rs4803455 in *TGFB1* is related to dental development age in individuals without CL ± P. Further studies should observe this association in other populations.

In the group with CL ± P, none of the SNPs evaluated was associated with variations in DA. The contrasting findings between the groups may be related to the fact that the expressivity of genotypes can be influenced by the accumulated impact of genetic variants and environmental factors [39]. CL ± P presents a complex etiology, involving genetic and environmental influences [40]. Therefore, it is reasonable to consider that individuals with and without CL ± P are under different expositions during their development, and consequently, may present differences in dental development. Moreover, other genetic variants could be associated with delayed DA in individuals with CL ± P. The lack of significant associations for the SNPs evaluated may reflect the complexity of genetic regulation in dental development and the potential functional differences among these genes in different phenotypic contexts. It is also possible that the small sample size prevented the detection of subtle genetic effects.

There is some evidence from studies with human and mouse embryos suggesting that the genes *EGF*, *EGFR*, and *TGFB2* have a function in dental development, playing a role in cell differentiation and proliferation, and influencing the formation of dental tissues [10, 11, 15, 17, 18, 33]. Studies in humans about the association between SNPs in these genes and dental development are still limited. Only the association between the rs4444903 in *EGF* with tooth size variations is reported in the literature [20]. In the present study, we did not find an association between the SNPs in these genes and DA variations.

Previous studies with individuals not affected by CL ± P explored the association between DA and SNPs in vitamin D receptor (*VDR*) [6], estrogen receptors alpha and beta (*ESR1* and *ESR2*, respectively) [22], and in the fibroblast growth factors (*FGF*) family [21]. SNPs in *VDR*, *ESR1*, and *ESR2* were not associated with DA variations [6, 22]. On the other hand, the SNP rs4073716 in the fibroblast growth factor 18 (*FGF18*) was associated with an advanced DA [21].

In addition to genetic factors, some environmental exposures, such as hormonal imbalances [41], obesity [42], celiac disease [43], and cancer therapy [44] are associated with variations in DA. In the present study, these expositions were not considered, which may be a limitation. The use of Demirjian’s method is frequently considered limited since it overestimates DA [27]. However, in the present study, we observed a strong correlation between DA and CA in both groups evaluated. The two groups evaluated were not matched by age here, but, we included children of similar ages and from the same geographical area, avoiding possible biases when comparing different groups [5]. Another limitation is related to the generalizability of our results. The findings of this study are from a specific population, which may limit their applicability to other populations with different genetic backgrounds or environmental conditions. Future studies should consider matching groups by age, exploring the interaction between environmental and genetic factors, and conducting longitudinal evaluations in different populations to better understand how DA progresses over time in patients with CL ± P.

Despite the limitations of this study, it was possible to observe that children with CL ± P present delayed DA compared with children without CL ± P. Clinically, this characteristic can influence adequate treatment timing since patients with CL ± P require innumerous interventions during their life, including lip and palate surgical repair, bone grafts, and orthodontic treatment [7]. Besides that, it can be a useful tool in the forensic field, serving as a complementary tool to maturity indicators for estimating the age of children with uncertain or unknown birth records [5]. The rs1800470 in TGFB1 was only associated with DA variation in the group of children without CL ± P, which may suggest that different factors influence dental development in the evaluated groups. Understanding the genetics underlying dental traits provides insight into the mechanisms of tooth development, serving as a valuable tool for identifying risk factors linked to tooth abnormalities. These findings emphasize the potential of integrating genetic and dental age assessments into clinical practice to optimize the management of children with CL ± P in the future, focusing on personalized treatment approaches and improving outcomes for patients with these craniofacial conditions.

**Conclusion**.

Children with CL ± P presented delayed DA compared with children without CL ± P. The SNP rs4803455 in *TGFB1* is associated with variations in DA in children without CL ± P. These findings contribute to a better understanding of craniofacial and dental development. Future studies should evaluate these variables in different populations, which may help enhance personalized care for patients with CL ± P.

## Electronic supplementary material

Below is the link to the electronic supplementary material.

Supplementary Material 1

Supplementary Material 2

## Data Availability

Data is provided upon reasonable request.

## Abbreviations

CA: Chronological age
CAIF: Centro de Atendimento Integral ao Fissurado Labiopalatal
CI: Confidence interval of 95%
CL: Cleft lip
CLP: Cleft lip and palate
CL ± P: Cleft lip with or without cleft palate
CP: Cleft palate
DA: Dental age
EGF: Epidermal Growth Factor
EGFR: Epidermal Growth Factor Receptor
ID: Identification
MD: Mean difference
MAF: Minor allele frequency
OR: Odds ratio
PCR: Polymerase chain reaction
SD: Standard deviation
SNP: Single nucleotide polymorphism
STREGA: Strengthening the Reporting of Genetic Association Study Statement Checklist
TGFB1: Transforming Growth Factor Beta 1
TGFBR2: Transforming Growth Factor Beta Receptor 2
UFPR: Federal University of Paraná
