# Supplementary material for: Polymorphisms and dental age in non-syndromic cleft lip and palate: a cross-sectional study
Source: BMC Pediatr. 2025 Jan 30;25:80. doi: 10.1186/s12887-025-05444-8 (PMC11783813; doi:10.1186/s12887-025-05444-8)
Supplement: Supplementary file 1 — Supplementary Material 1 [file 12887_2025_5444_MOESM1_ESM.docx]

| Additional File 1 Table S1 – Distribution of the observed frequency of genotype and Hardy-Weinberg equilibrium of the genetic polymorphisms evaluated in this study (n=198). | | | | | | | | |
| --- | --- | --- | --- | --- | --- | --- | --- | --- |
| Gene | **SNP** | **Group** | **Genotype (n%)** | | | **Total** | **Genotyping success rate** | **Chi-squared HWE** |
|  |  |  | **AA** | **AG** | **GG** |  |  |  |
| *EGF* | rs4444903 | With CL±P | 26 (28.3) | 48 (52.2) | 18 (19.6) | 92 | 98.9% | 0.1022 |
|  |  | Without CL±P | 23 (21.9) | 55 (52.4) | 27 (25.7) | 105 | 100% | 0.2536 |
|  |  |  | **AA** | **AG** | **GG** |  |  |  |
|  | rs2237051 | With CL±P | 19 (20.4) | 43 (46.2) | 31 (33.3) | 93 | 100% | 0.3305 |
|  |  | Without CL±P | 24 (22.9) | 57 (54.3) | 24 (22.9) | 105 | 100% | 0.6053 |
| *EGFR* |  |  | **AA** | **AG** | **GG** |  |  |  |
|  | rs2227983 | With CL±P | 8 (8.7) | 29 (31.5) | 55 (59.8) | 92 | 98.9% | 1.9859 |
|  |  | Without CL±P | 8 (7.7) | 35 (33.7) | 61 (58.7) | 104 | 99.0% | 0.8574 |
| *TGFB1* |  |  | **CC** | **CA** | **AA** |  |  |  |
|  | rs4803455 | With CL±P | 33 (35.9) | 47 (51.1) | 12 (13.0) | 92 | 98.9% | 0.5583 |
|  |  | Without CL±P | 40 (39.2) | 49 (48.0) | 13 (12.7) | 102 | 97.1% | 0.1123 |
|  |  |  | **AA** | **AG** | **GG** |  |  |  |
|  | rs1800470 | With CL±P | 21 (22.8) | 49 (53.3) | 22 (23.9) | 92 | 98.9% | 0.3928 |
|  |  | Without CL±P | 19 (18.4) | 54 (52.4) | 30 (29.1) | 103 | 98.1% | 0.3788 |
| *TGFBR2* |  |  | **CC** | **CG** | **GG** |  |  |  |
|  | rs764522 | With CL±P | 52 (55.9) | 36 (38.7) | 5 (5.4) | 93 | 100% | 0.147 |
|  |  | Without CL±P | 64 (62.7) | 32 (31.4) | 6 (5.9) | 102 | 97.1% | 0.5395 |
|  |  |  | **AA** | **AG** | **GG** |  |  |  |
|  | rs3087465 | With CL±P | 7 (7.6) | 41 (44.6) | 44 (47.8) | 92 | 98.9% | 0.3684 |
|  |  | Without CL±P | 15 (15.0) | 41 (41.0) | 44 (44.0) | 100 | 95.2% | 1.0963 |
| Abbreviations: CL±P – Non-syndromic cleft lip with or without palate; *EGF* – Epidermal Growth Factor; *EGFR* – Epidermal Growth Factor Receptor; *TGFB1* – Transforming Growth Factor Beta 1; *TGFBR2* – Transforming Growth Factor Receptor 2. | | | | | | | | |
